# Enhanced Endoscopic Imaging Technologies in Upper Urinary Tract Urothelial Tumors: Implications for Kidney-Sparing Surgery—A Narrative Review

**DOI:** 10.3390/diagnostics16142187

**Published:** 2026-07-14

**Authors:** Lucía Polanco-Pujol, Jorge Caño-Velasco, Mario Tapia-Tapia, Guillermo Barbas-Bernardos, Luis Labairu-Huertas, Daniel Sánchez-Zalabardo, Felipe Villacampa-Aubá, Daniel González-Padilla, Francisco-Javier Ancizu-Marckert, Fernando Diez-Caballero-Alonso, José Enrique Robles-García, José Daniel Subiela, Bernardino Miñana-López

**Affiliations:** 1 Department of Urology, Clínica Universidad de Navarra, Cancer Center, 31008 Pamplona, Spain; lpolancopuj@unav.es (L.P.-P.); jcanovelasc@unav.es (J.C.-V.); mdtapia@unav.es (M.T.-T.); llabairu@unav.es (L.L.-H.); dsanchezz@unav.es (D.S.-Z.); fancizu@unav.es (F.-J.A.-M.); fdcaballero@unav.es (F.D.-C.-A.); 2 Department of Urology, Clínica Universidad de Navarra, Cancer Center, 28027 Madrid, Spain; gbarbasb@unav.es (G.B.-B.); fvauba@unav.es (F.V.-A.); dgonzalezp@unav.es (D.G.-P.); 3 Department of Urology, Hospital Universitario Ramón y Cajal, 28034 Madrid, Spain; jdsubiela@gmail.com

**Keywords:** upper tract urothelial carcinoma, ureterorenoscopy, image-enhanced endoscopy, photodynamic diagnosis, narrow band imaging, optical coherence tomography, confocal laser endomicroscopy, endoluminal ultrasonography

## Abstract

Upper tract urothelial carcinoma (UTUC) represents 5–10% of urothelial malignancies and poses significant diagnostic challenges. Ureterorenoscopy (URS) remains essential for definitive diagnosis, tumor characterization, and selection of candidates for kidney-sparing surgery. Recent advances in endoscopic image-enhancement technologies aim to overcome the limitations of conventional white-light URS. The aim of this study is to review the current evidence on advanced endoscopic imaging techniques for the diagnosis and management of UTUC. This narrative literature review summarizes current evidence on advanced endoscopic imaging techniques for UTUC. A comprehensive search of PubMed/MEDLINE, EMBASE, and Scielo databases was conducted to identify studies evaluating photodynamic diagnosis (PDD), narrow band imaging (NBI), Image1-S™-Spies™, optical coherence tomography (OCT), confocal laser endomicroscopy (CLE), and endoluminal ultrasonography (ELUS). Data were qualitatively synthesized by modality. Macroscopic enhancement techniques (PDD, NBI, Image1-S™) improve visualization of urothelial surfaces and vascular patterns during URS. PDD demonstrates the highest diagnostic accuracy, with sensitivities up to 95.8%, and a pooled sensitivity and specificity of 0.96 and 0.86. NBI increases tumor detection rates, identifying up to 22.7% additional lesions in early studies. Microscopic imaging modalities provide complementary information. OCT enables high-resolution cross-sectional imaging with 93% concordance with pathological staging. CLE offers in vivo optical biopsy with near-histological resolution, achieving concordance rates of 100% for low-grade and 83–86% for high-grade tumors. ELUS allows deeper tissue assessment but with lower spatial resolution. Enhanced endoscopic imaging technologies are promising, improving tumor detection, characterization, and intraoperative risk stratification. However, current evidence remains limited and heterogeneous, highlighting the need for large prospective multicenter studies to define their impact on oncological outcomes.

## 1. Introduction

Upper tract urothelial carcinomas (UTUC) represent only 5–10% of urothelial carcinomas. Historically, radical nephroureterectomy (RNU) with bladder-cuff excision has been considered the standard treatment. However, kidney-sparing surgery (KSS) may be considered a valid alternative management in selected cases in order to maintain renal function without compromising oncological safety [1].

Although computed tomography urography (CT) provides the highest diagnostic accuracy among imaging modalities, ureterorenoscopy (URS) remains essential for definitive diagnosis, enabling direct visualization of the upper urinary tract (UUT), tumor biopsy, and, in selected cases, endoscopic treatment [2,3]. Current clinical practice guidelines consider KSS in patients with “low-risk UTUC”, characterized by all of the following: unifocal disease, tumor size < 2 cm, negative for high-grade cytology, low-grade URS biopsy, and no invasive aspect on CT scan [1].

Optimal endoscopic visualization is crucial for reliable assessment of tumor characteristics and for performing accurate biopsy and complete tumor ablation [2,4]. Several factors have improved the endoscopic diagnosis and treatment of UTUC in recent years, including improvements in digital imaging, laser technology, enhanced miniaturization of materials, and image enhancement technologies [2,5], such as narrow band imaging (NBI), photodynamic diagnosis (PDD), and the Image1-S™-Spies™ system (Karl Storz^®^, Tuttlingen, Germany), among others. Originally developed to enhance cystoscopic vision in bladder cancer, these technologies have been incorporated into flexible URS (f-URS) for UTUC to overcome the limitations of white-light (WL) URS and improve detection, sensitivity (Se), and specificity (Sp), particularly for flat lesions such as carcinoma in situ (CIS) [2,6,7]. Current clinical practice guidelines generally consider image enhancement technologies as promising adjuncts for the evaluation of UTUC, but not part of standard clinical practice. The available evidence is limited and largely based on small retrospective studies focusing on diagnostic performance, without demonstrated impact on oncological outcomes [1].

The aim of our study was to perform a narrative review of the available literature evaluating enhanced imaging techniques for the UTUC diagnosis and/or treatment.

## 2. Materials and Methods

### 2.1. Study Design

A narrative literature review was conducted following the PRISMA criteria to summarize the current evidence regarding advanced endoscopic imaging technologies for the diagnosis and treatment of UTUC. The review focused on macroscopic and microscopic image-enhancement modalities applied during URS, including photodynamic diagnosis (PDD), narrow band imaging (NBI), Image1-S™-Spies™, optical coherence tomography (OCT), confocal laser endomicroscopy (CLE), and endoluminal ultrasonography (ELUS).

### 2.2. Search Strategy

A comprehensive literature search was performed in PubMed/MEDLINE, EMBASE, and Scielo databases to identify relevant studies published between January 2010 and January 2026.

The search strategy combined Medical Subject Headings (MeSH) and free-text keywords related to UTUC and image enhancement technologies. The main search terms included: “upper urinary tract”, “upper tract urothelial carcinoma”, “ureteral cancer”, “renal pelvis tumor”, “ureteroscopy”, “endoscopic imaging”, “image enhancement”, “photodynamic diagnosis”, “narrow band imaging”, “SPIES”, “optical coherence tomography”, “confocal laser endomicroscopy”, and “endoluminal ultrasonography”.

Boolean operators (AND/OR) were used to combine terms when appropriate. The search was limited to articles published in English involving human subjects.

### 2.3. Study Selection

Two independent reviewers screened all titles and abstracts retrieved from the search to identify potentially relevant studies. Full-text evaluation was subsequently performed for eligible articles. Studies were included if they met the following criteria:

#### 2.3.1. Inclusion Criteria

• Studies evaluating endoscopic imaging technologies applied to UTUC.
• Studies assessing diagnostic performance, tumor detection, staging, or oncologic outcomes.
• Prospective or retrospective clinical studies.
• Systematic reviews and meta-analyses.
• Experimental or translational studies related to optical diagnostic technologies in the UUT.

#### 2.3.2. Exclusion Criteria

• Studies not related to UTUC.
• Articles focusing exclusively on bladder cancer without relevance to the UUT.
• Non-English publications.
• Conference abstracts without full text.
• Duplicate publications.

Any discrepancies between the two reviewers were resolved by discussion and, when necessary, consultation with a third reviewer.

### 2.4. Data Extraction and Synthesis

Data extraction was performed independently by two investigators and subsequently verified for accuracy. Given the heterogeneity of study designs, imaging technologies, and reported outcomes, a qualitative synthesis of the evidence was performed rather than a quantitative meta-analysis.

Studies were grouped according to the imaging modality investigated, and results were summarized focusing on the following:

• Diagnostic performance.
• Advantages and limitations.
• Clinical applicability during URS.
• Potential impact on KSS strategies.

**Figure 1:**
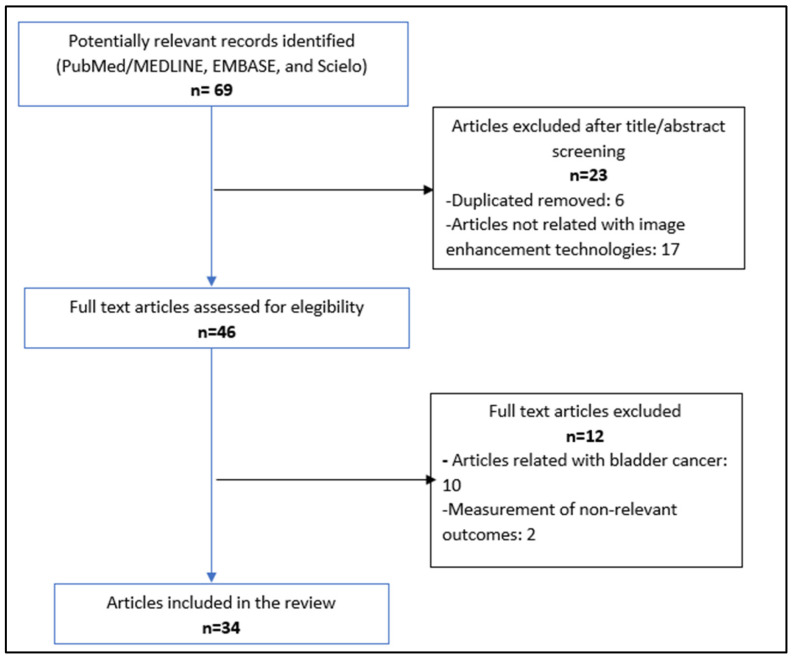
Flow diagram of study selection.

The study selection process is summarized in Figure 1, illustrating the PRISMA diagram with the number of studies identified, screened, excluded, and finally included in the scoping review.

### 2.5. Potential Bias

The studies included were critically evaluated regarding potential sources of bias. The main limitations identified across the literature were selection bias, small sample sizes, heterogeneous study designs, publication bias, and differences in outcome definitions and measurement methods. These factors should be considered when interpreting the findings.

## 3. Results and Discussion

### 3.1. Photodynamic Diagnosis (PDD)

Originally introduced for bladder cancer detection [8], PDD was subsequently extended to URS as a diagnostic and therapeutic tool in UTUC [9]. PDD employs oral 5-aminolevulinic acid (5-ALA) as a photosensitizer prior to URS, usually at a dose of 20 mg/kg. A total of 5-ALA is metabolized into protoporphyrin IX (PpIX), which preferentially accumulates in malignant urothelial cells and fluoresces under blue-violet light (375–445 nm), enhancing tumor visualization during endoscopy [6,7,10,11,12]. In comparison, for the diagnosis of bladder cancer, 5-ALA can be administered either orally or intravesically prior to the cystoscopy, whereas hexaminolevulinate (HAL) is used exclusively via intravesical instillation [13]. HAL, as a lipophilic ester of 5-ALA, exhibits improved solubility, higher local bioavailability, and an increased PpIX formation at lower doses compared with 5-ALA [13].

Although the diagnostic potential of PDD for UTUC has been evaluated in various studies, clinical approval for its use in this setting has not been established. PDD may play a crucial role in cases where lesions are not visibly detectable (flat lesions, CIS, dysplasia), since random biopsies—commonly performed in the bladder—are often not feasible in the UUT [14,15,16]. However, the use of PDD can cause adverse effects such as photosensitive skin rashes, transient hypotension, and alterations in liver enzyme levels, which have been observed in up to 25% of patients [17].

Kata et al., in a large case series (*n* = 106), demonstrated that PDD with f-URS D-Light C^®^ (Karl Storz^®^, Tuttlingen, Germany) was associated with higher Se (95.8% vs. 53.5%; *p* < 0.0001) and tumor detection rates (TDR) (93.7% vs. 18.7%; *p* = 0.0006) compared with WL-f-URS [15]. Similar high Se has also been reported in other studies [18,19,20,21,22].

Subsequently and in line with the above, a systematic review and meta-analysis published in 2020 demonstrated that PDD is highly effective in distinguishing UTUC from benign lesions, with a Se of 0.96 (95% CI: 0.85–0.99) and Sp of 0.86 (95% CI: 0.64–0.95; area under curve [AUC]: 0.97). Moreover, in comparison to WL-URS, PDD significantly improves the detection rate of UTUC (Relative Risk [RR]: 0.16, 95% CI: 0.07–0.34, *p* = 0.000) [23]. A recent retrospective study showed that PDD with oral 5-ALA had a higher AUC than WL in suspected UTUC undergoing URS (0.84 vs. 0.74) and in suspected bladder cancer undergoing transurethral resection of the bladder tumor (TURBT) (0.73 vs. 0.69). Although the overall performance was higher in the PDD-URS cohort than in PDD-TURBT, the results were comparable to those of the PDD-TURBT-naïve subgroup (0.84 vs. 0.76). On the other hand, PDD may be associated with false positives (FP), particularly due to local inflammation [17]. Sano et al. reported that the incidence of precancerous findings in FP PDD samples was higher in the PDD-URS cohort than in the PDD-TURBT cohort (57.1% vs. 7.9%, *p* = 0.00028) [16].

A prospective non-randomized study evaluated the usefulness of PDD (5-ALA) to diagnose UTUC using the VISERA ELITE Video System (Olympus Co., Ltd., Tokyo, Japan), showing that while this image system may not be fully effective for detecting UTUC, it demonstrates higher Se for identifying CIS compared to WL [12].

Current evidence on the oncological outcomes of PDD in the UUT is very limited. In contrast, several studies have reported varying results regarding PDD in bladder cancer. A Cochrane review (16 randomized controlled trials [RCTs]; *n* = 4.325) indicated that PDD-TURBT may delay both recurrence and progression compared to WL-TURBT [24]. Conversely, a multicenter RCT showed that PDD-TURBT offered no benefit in recurrence reduction and was not cost-effective compared with WL cystoscopy [25]. In 2025, Soria et al. published a document intended to standardize the eligibility criteria for PDD in non-muscle-invasive bladder cancer (NMIBC) across different clinical scenarios, in line with current international guideline recommendations [26].

Yoshida et al. compared the efficacy of the PDD-guided dual laser ablation (PDD-DLA) technique for UTUC using thulium:YAG and holmium (Ho):YAG lasers with conventional Ho:YAG laser ablation (HLA) as a historical control [27]. PDD-DLA was associated with a significantly higher 2-year progression-free survival (PFS) rate (100% vs. 58.7%, *p* = 0.0197) and a higher 2-year recurrence-free survival (RFS) rate (57.1% vs. 41.3%, *p* = 0.072). Furthermore, the PDD-DLA group showed a significantly lower incidence of salvage RNU compared to the HLA group (0.0% vs. 50%; *p* = 0.009). In addition, the FLUAM trial is a prospective, single-center, single-arm pilot study designed to evaluate the efficacy and safety of PDD-URS (5-ALA) laser ablation in patients with localized UTUC, with the primary endpoint being PFS, and whose results have not yet been published [28].

Although the current evidence regarding PDD in UTUC remains limited and its implementation poses significant technical challenges, this technique shows promising potential as a complementary tool to conventional URS for UUT evaluation. However, its clinical application is still constrained by technical limitations, the limited availability of dedicated ureteroscopes, the risk of FP findings resulting from tangential viewing angles, high costs, and the lack of robust evidence regarding its impact on tumor recurrence and progression [6,7,10,29].

### 3.2. Narrow Band Imaging (NBI)

**Figure 2:**
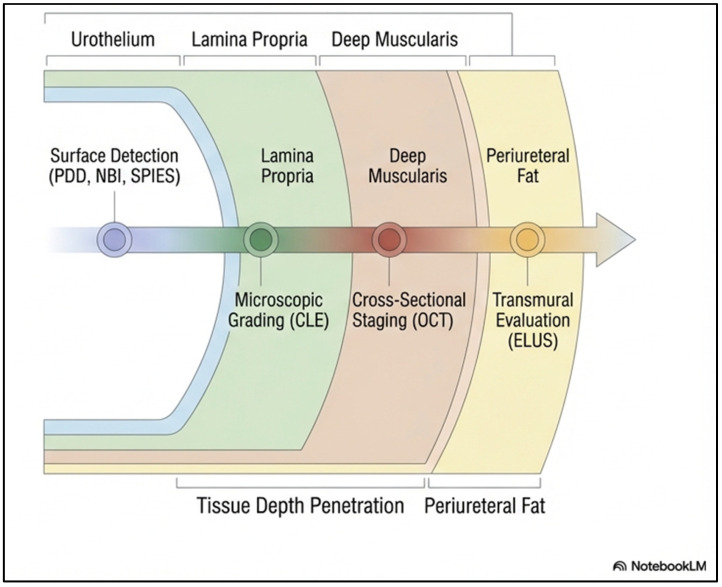
Penetration depth of enhanced endoscopic imaging technologies. Source: This image was generated using NotebookLM Version web (Google, Mountain View, CA, USA) based on author-provided prompts and subsequently reviewed and edited by the authors.

Color perception depends on the wavelength of light: blue, green, and red correspond approximately to 400, 550, and 600 nm, respectively, whereas WL contains a broad spectrum (400–700 nm). The narrower the spectrum, the greater the intensity and brightness of the perceived color. The NBI system uses a filter that transforms the WL emitted by the endoscope (with a broad wavelength spectrum) into light with two narrow bands of 415 nm (blue) and 540 nm (green). These wavelengths are selectively absorbed by hemoglobin, allowing blood vessels to stand out in contrast to the surrounding mucosa [13,30]. In contrast to PDD, instillation of an exogenous contrast agent is not required. Furthermore, due to the varying tissue penetration of different wavelengths, blue light—with its shorter wavelength—emphasizes the superficial vasculature, while green light—with a longer wavelength—enhances visualization of deeper mucosal and submucosal vessels [13,30] (Figure 2).

NBI has been well described for bladder urothelial carcinoma [31]; however, published experience in the UUT remains limited. NBI is available either as an integrated video cystoscope or as a camera head that can be fitted to a telescope, enhancing the visualization of the urothelium and its microvasculature. The vasculature is highlighted, appearing dark green to black, while the normal mucosa appears pale white. This contrast facilitates the identification of neovascularization and delineates the boundaries between vascularized and non-vascularized structures. The enhanced contrast improves the detection and characterization of small and flat lesions (CIS) that may be overlooked under WL imaging; however, distinguishing between flat and papillary lesions can sometimes be challenging with NBI. Image quality may be suboptimal in the presence of hematuria or inflammation, and NBI should always be combined with WL cystoscopy to minimize FP findings.

There is no significant learning curve associated with NBI. Studies have shown that after a single training and observation session, a comparable number of additional urothelial carcinomas can be detected compared to those identified by experienced operators. Furthermore, interobserver variability is minimal, and detection rates do not differ between expert and novice users [32].

Several studies have evaluated the diagnostic performance of NBI for bladder urothelial carcinoma compared with conventional WL imaging, consistently demonstrating higher TDR. A meta-analysis published by Zheng et al. (*n* = 1022 patients) demonstrated significant improvements in per-patient diagnostic performance with NBI cystoscopy compared with conventional WL cystoscopy, with a higher Se (94.3% vs. 84.8%) and similar Sp (84.7% vs. 87%). The AUC was 0.97 for NBI vs. 0.89 for WL. For CIS detection, NBI achieved a Se of 92.7% and Sp of 76.8%, with an AUC of 0.94 [33]. Xiong et al. (*n* = 1557) showed that NBI cystoscopy significantly outperformed WL cystoscopy in NMIBC, with higher additional TDR per patient (9.9%) and per lesion (18.6%), particularly for CIS (25.1% and 31.1%, respectively). NBI showed superior Se at both patient and lesion levels and was associated with a significantly reduced recurrence risk (RR: 0.43). These results support NBI as a more effective tool than conventional cystoscopy for detecting NMIBC and CIS and for decreasing recurrence [34].

Traxer et al. were among the first to apply this technique to the UUT in 2011. NBI resulted in a 22.7% improvement in TDR. They evaluated the UUT of 27 patients (14 with known UTUC and 13 with first suspicion of cancer) using both WL and NBI. Five additional tumors were identified in four patients (14.2%), and three tumors (8.5%) showed a wider extent when compared with WL [35].

Other series, such as that of Iordache et al. [36], also report improvements in diagnostic performance. In their prospective analysis (*n* = 87) with primary suspected UTUC, a digital f-URS with a biopsy of the visible lesions was performed using both WL and NBI. The patient TDR was significantly higher with NBI compared with the standard f-URS (98.4% vs. 91.9%; *p* < 0.05), identifying more exclusive urothelial carcinoma cases (8.1% vs. 1.6%) and additional renal transitional cell lesions (12.9% vs. 1.6%). These findings are consistent with reports showing significantly higher overall detection rates for overall UTUC and pTa tumors with NBI f-URS compared with WL evaluation (98.2% vs. 86.7%; *p* < 0.05 and 98.1% vs. 87.5%; *p* < 0.05, respectively). Although the number of CIS lesions was relatively small, NBI virtually confirmed a superior diagnostic performance over WL (100% vs. 77.8%), without statistical significance (*p* > 0.05). Furthermore, the rate of FP results was significantly higher for NBI compared with WL (17.5% vs. 10.1%; *p* < 0.05).

Recent studies, such as that by Geavlete et al., have focused on the usefulness of NBI during the conservative management of UTUC, with the aim of evaluating recurrence rates in conservatively treated cases [37]. The use of NBI allows for a more accurate identification of tumor margins, enabling complete lesion resection and thereby reducing the likelihood of residual disease. They compared NBI-assisted visualization during diagnosis and Ho laser treatment with a control group treated without NBI, in a cohort of 61 patients with superficial pyelocaliceal urothelial tumors. After one year, recurrence rates were lower in the NBI group (3.3% vs. 8.2%, *p* < 0.05); in low-grade (LG) tumors, the recurrence was 1.8%, while in high-grade (HG) tumors it reached 20%. At three years, recurrence remained reduced with NBI (11.5% vs. 18%, *p* < 0.05), including both LG (7.1% vs. 21.4%) and HG tumors (40% vs. 100%, *p* < 0.05). Cancer-specific survival (CSS) was also higher in the NBI group (93.4% vs. 86.9%, *p* < 0.05). Overall, NBI use during URS and laser vaporization significantly decreased recurrence rates and improved CSS compared with standard techniques.

Regarding bladder cancer, multiple meta-analyses have shown a significant reduction in recurrence rates with the use of NBI-assisted TURBT in both the short and long term. Xiong et al. [34] showed that NBI significantly decreased bladder cancer recurrence, with a pooled RR of 0.43 (95% CI: 0.23–0.79) at three months and 0.81 (95% CI: 0.69–0.95) at twelve months. Additionally, Kang et al. [38] reported a one and two-year relative risk of recurrence of 0.52 (95% CI, 0.40–0.67) and 0.60 (95% CI, 0.42–0.85), respectively, compared with WL-TURBT. These observations may be cautiously extrapolated to tumors of UUT, suggesting that NBI-guided interventions might facilitate conservative, organ-preserving approaches, while maintaining oncological safety in this setting remains to be fully established. Nevertheless, despite promising diagnostic performance, evidence demonstrating a clear impact on long-term oncological outcomes remains limited.

Compared with PDD, NBI offers several practical advantages, including the absence of photosensitizing agents, facilitating its integration into routine clinical practice. Although PDD may provide higher Se, particularly for CIS detection, the need for photosensitizer administration is associated with increased procedural costs. In contrast, NBI provides immediate applicability, lower costs, and greater operational simplicity, which could support its widespread use in urothelial cancer assessment.

### 3.3. Image1-S™-Spies™ System

In addition to conventional WL imaging, the Image1-S™-Spies™ system (Karl Storz^®^, Tuttlingen, Germany) incorporates five distinct visual enhancement modes. Images are initially acquired under WL using an RGB (red, green, and blue) camera and subsequently undergo digital post-processing to generate the selected enhanced imaging modality [39,40]: (a) Clara, which optimizes brightness to achieve better views of dark spots; (b) Chroma, which increases color contrast to highlight tissue differences; (c) Clara + Chroma, combining both effects; and (d) Spectra A and B, post-processing virtual chromoendoscopy enhancements that use spectral filters that alter color representation to emphasize surface patterns and vascular structures, thereby improving visualization of the mucosa and lesions during URS. The original WL image and the enhanced mode are displayed side by side on the endoscopy monitor in real time, in contrast to PDD or NBI [13].

The Image1-S™-Spies™ system enhancement modes have been graded significantly better than conventional WL imaging for bladder urothelium visualization [41], potentially allowing improved diagnostic accuracy, a reduced risk of false-negative findings, and increased detection of suspicious lesions [42,43,44]. Recurrence rates at 12–18 months post-TURBT have been reported to be similar between Image1-S™ and WL guidance; however, Image1-S™-assisted TURBT was associated with significantly lower recurrence rates in patients with primary, low- to intermediate-risk NMIBC [45,46]. Its role in progression rates, however, is not yet well established [13].

Although several studies have evaluated the Image1-S™-Spies™ system in bladder cancer, scientific evidence regarding its application in the UUT remains limited. In 2017, Emiliani et al. compared the URS image quality of five imaging modalities (Spies™ system integrated in the FlexXC™ URS) with standard WL in an in vitro experimental model, obtaining better subjective image quality for the Clara and Clara + Chroma modes compared to WL (*p* = 0.0139 and *p* < 0.05, respectively) and worse for Spectra A and B (*p* = 0.0005 and *p* = 0.0023, respectively). In comparison with each other, Clara and Clara + Chroma showed equivalent performance (*p* = 0.67), and both outperformed Chroma alone, Spectra A, and Spectra B [40]. To date, no study has evaluated whether this improvement could affect the TDR during URS [5].

The image quality of D-URS is superior to that achieved with fiber-optic URS (FO-URS) [47]. The Clinical Research Office of the Endourology Society (CROES)-UTUC registry analyzed 401 patients who underwent URS (186 FO-URS and 215 D-URS). No significant differences were observed between the two URS modalities in overall survival (OS) (*p* = 0.9) or disease-free survival (DFS) (*p* = 0.7). In addition, the use of NBI (*n* = 64, 2.7%) and Image1-S™ (*n* = 94, 3.9%) technologies in D-URS cases did not result in improvements in OS (*p* = 0.5) or DFS (*p* = 0.3). These findings suggest that, although advanced imaging modalities improve visualization, their direct impact on long-term oncological outcomes remains to be fully established [42].

Image1-S™ may offer advantages over PDD and NBI by avoiding intravesical agents (HAL or 5-ALA) and specialized equipment, reducing procedure time and costs, and allowing real-time side-by-side comparison of WL and enhanced images. However, further trials are needed to define its position relative to PDD and NBI [13].

Table 1 shows characteristics of PDD, NBI, and the Spies™ system for UTUC.

### 3.4. Optical Coherence Tomography (OCT)

**Figure 3:**
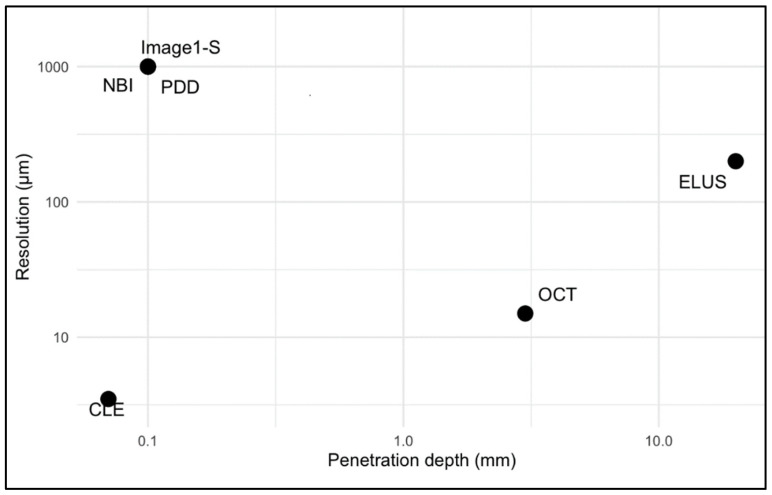
Resolution and penetration depth of enhanced endoscopic imaging technologies for UTUC.

OCT is a non-invasive imaging modality based on near-infrared light and low-coherence interferometry, capable of generating high-resolution cross-sectional images of biological tissues [48]. It is frequently described as the optical equivalent of ultrasound (US); however, by measuring backscattered light rather than sound waves, OCT achieves substantially higher spatial resolution [48,49]. OCT provides an axial resolution in the range of 10–20 μm (Figure 3), which is approximately 10 to 20 times higher than that of high-frequency US. In practical terms, this resolution allows clear delineation of the urothelium, lamina propria, and muscularis propria under normal conditions [48,49]. While sufficient for evaluating superficial layers, this constraint may prevent accurate visualization of the deepest invasion front in bulky or markedly exophytic tumors (penetration depth of 2–3 mm) [48,49,50]. Beyond morphological staging, OCT also offers quantitative information through the calculation of the optical attenuation coefficient (μOCT), reflecting the exponential decay of the signal within tissue [51].

Evidence from the NOCTURN study indicates that OCT-based T staging (octT) achieved a 93% concordance rate with final pathological staging when differentiating non-muscle invasive disease (pTis/Ta/T1) from muscle-invasive disease (T2/T3) [50]. This performance notably exceeds that of conventional clinical diagnostic and radiographic imaging (concordance of 79%) [50]. Moreover, in small lesions, OCT demonstrated 100% Se for detecting tumor invasion, highlighting its value in early-stage disease [48]. Furthermore, quantitative grading using the μOCT with a cut-off value of >4.0 mm^−1^ has shown a Se of 83% and a Sp of 94% for identifying HG papillary UTUC [51]. Regarding CIS, evidence remains limited (*n* = 2); in the NOCTURN study, diagnostic accuracy for pTis lesions was 50% [50].

Accurate intraoperative staging provided by OCT can directly influence therapeutic decision-making, particularly when considering KSS. Given that ≥T2 disease generally mandates RNU with lymph node dissection, reliable differentiation from non-muscle-invasive disease is critical [50]. Additionally, during follow-up, OCT may also reduce the need for repeated biopsies by enabling real-time assessment of previously treated areas, although long-term outcome data remain limited [49].

### 3.5. Confocal Laser Endomicroscopy (CLE)

CLE is commonly referred to as a “real-time optical biopsy” as it enables in vivo microscopic visualization of the urothelium during URS [52,53,54]. Unlike cross-sectional imaging modalities, CLE provides near-histological resolution at the cellular level. The most widely used system (Cellvizio^®^; Mauna Kea Technologies, Paris, France) operates with a 488 nm laser and requires fluorescein—administered either topically or intravenously—as a contrast agent to enhance visualization of the extracellular matrix and cellular architecture [52]. Common protocols use 3–5 mL of 0.1% fluorescein through the working channel of the URS with a 3–5 min dwell time before imaging [52,53,55], while some studies employ 0.5 mL of 2.5% for immediate acquisition [54,56]; however, image quality only typically lasts about 5 min due to rapid urinary washout [52,55].

CLE provides high lateral resolution (~3.5 μm), enabling near-histologic visualization of cellular morphology and architecture, but its limited imaging depth (40–70 μm) and a lateral field of view of approximately 325 μm, restrict assessment to superficial tissues without evaluating deeper stromal invasion (Figure 2) [52,53,54,55].

Characteristic patterns on CLE have been described for tumor grading. LG lesions generally demonstrate organized, monomorphic cellular architecture with identifiable fibrovascular stalks. In contrast, HG tumors display architectural disarray, loss of cellular polarity, pleomorphism, and irregular, tortuous vascular patterns. CLE shows a strong correlation with final histopathology, reporting concordance rates that reach 100% for LG tumors and 83–86% for HG tumors [53,54,55]. Se and Sp levels for LG have been reported at 79% and 78%, respectively; while HG show a Se of 67% and a Sp of 79%, with a substantial interobserver agreement (K = 0.64) [53,55]. Other studies show that CLE demonstrates a Se of 100% for HG disease, although its Sp may be limited to 57.1% due to potential FP caused by inflammatory or reactive changes [57].

Specifically for CIS, CLE allows for the identification of pleomorphic cells and disorganized microarchitecture in flat lesions that are frequently indistinguishable from benign inflammation under conventional endoscopy. Breda et al. reported a 100% concordance rate (1 out of 1 case) between in vivo evaluation and the final histopathological results [53]. To further reduce interobserver variability, scoring systems incorporating parameters such as cellular organization and cohesiveness have been proposed to formalize real-time grading [54].

From a practical standpoint, CLE enables immediate intraoperative risk stratification. When LG disease is confirmed, tumor ablation can be performed during the same URS session, potentially reducing the need for staged procedures [53,55]. Contemporary cohorts combining CLE with image enhancement technologies have reported kidney preservation rates of 82.9% [57].

### 3.6. Endoluminal Ultrasonography (ELUS)

ELUS utilizes high-frequency sound waves, typically ranging from 20 to 30 MHz, delivered through a small mechanical radial scanning probe approximately 1.7 mm (5F) in diameter [58,59]. The probe is designed to be advanced over a guidewire into the UUT under fluoroscopic guidance. This technique provides a circumferential (360°) cross-sectional view of the ureteral wall and adjacent anatomical structures in B-mode, offering a perspective that complements optical imaging modalities like OCT and CLE [48,49,58].

Compared with OCT, ELUS has a significantly lower axial resolution of approximately 200 μm [48,49,59]. While this allows for the visualization of major anatomical layers, such as the hypoechoic muscularis and hyperechoic periureteral fat, it is insufficient to reliably differentiate the thin urothelium from the lamina propria, limiting its capacity for precise superficial staging [48,49]. The principal advantage of ELUS lies in its substantial penetration depth, which can reach up to 20 mm (Figure 2) [48,59]. This capability allows for the assessment of structures well beyond the ureteral wall, including periureteral tissues, regional lymph nodes, and crossing vessels—anatomical areas that remain inaccessible to the limited range of OCT or CLE [48,49].

Beyond oncologic staging, ELUS has shown high effectiveness in confirming non-muscle-invasive disease, with initial pilot studies reporting a negative predictive value of 100% [48,58]. However, expanded cohorts have revealed that its positive predictive value for muscle-invasive disease (≥pT2–T3) remains low, approximately 16.7% [48,59]. Overstaging frequently occurs in the presence of inflammatory wall thickening, edema, or bulky lesions where resolution constraints prevent the surgeon from accurately discriminating between tumor mass and the underlying muscular layers. Consequently, ELUS findings must be carefully interpreted within the broader clinical and endoscopic context [48,59].

Table 2 shows characteristics of OCT, CLE, and ELUS imaging technologies for UTUC.

A summary of the advantages and limitations of enhanced endoscopic imaging technologies is presented in Table 3.

## 4. Conclusions

Enhanced endoscopic imaging technologies improve visualization and diagnostic accuracy in UTUC, with potential benefits for tumor detection, staging, and intraoperative risk stratification. These advances may facilitate a more appropriate selection of patients for kidney-sparing management. However, despite encouraging diagnostic performance, evidence demonstrating a clear impact on long-term oncological outcomes remains limited.

Moreover, the widespread adoption of these technologies is constrained by factors such as limited availability, high costs, technical complexity, and the lack of standardized training. Current evidence is also largely based on small, heterogeneous studies.

Therefore, the future of UTUC management will likely rely on a multimodal, risk-adapted approach, as no single technology is sufficient. Well-designed prospective, multicenter studies are needed to better define their clinical role, assess cost-effectiveness and patient-centered outcomes, and explore the integration of artificial intelligence to further enhance diagnostic accuracy and support clinical decision-making.

## Figures and Tables

**Table 1:** Characteristics of macroscopic system imaging technologies for UTUC.

Technique	Principle	Contrast Required	Resolution	Penetration Depth	Main Clinical Application
Photodynamic diagnosis (PDD)	Fluorescence after 5-ALA metabolism under blue light	Yes (5-ALA/HAL)	Macroscopic	Superficial mucosa	Detection of flat lesions and CIS
Narrow band imaging (NBI)	Selective light wavelengths absorbed by hemoglobin	No	Macroscopic	Superficial mucosa	Vascular pattern enhancement
Image1-S (SPIES)	Digital post-processing spectral enhancement	No	Macroscopic	Superficial mucosa	Improved mucosal visualization

**Table 2:** Characteristics of OCT, CLE, and ELUS imaging technologies for UTUC.

Technique	Principle	Contrast Required	Resolution	Penetration Depth	Main Clinical Application
Optical coherence tomography (OCT)	Near-infrared interferometry cross-sectional imaging	No	10–20 μm	2–3 mm	Tumor staging
Confocal laser endomicroscopy (CLE)	Confocal microscopy cellular imaging	Yes (fluorescein)	~3.5 μm	40–70 μm	Real-time optical biopsy
Endoluminal ultrasonography (ELUS)	High-frequency ultrasound radial probe	No	~200 μm	Up to 20 mm	Assessment of deep invasion

**Table 3:** Advantages and limitations of enhanced endoscopic imaging technologies for UTUC.

Technique	Main Advantages	Limitations
PDD	High sensitivity for CIS and flat lesions	Photosensitivity reactions, false positives with inflammation, and higher cost
NBI	No contrast agents required, easy integration into endoscopic systems	Reduced image quality in hematuria or inflammation
SPIES	Real-time digital enhancement and side-by-side visualization	Limited clinical evidence in UTUC
OCT	High-resolution cross-sectional imaging for staging	Limited penetration depth
CLE	Real-time microscopic imaging (optical biopsy)	Limited field of view and superficial penetration
ELUS	Deep tissue penetration and evaluation of periureteral structures	Low spatial resolution for superficial layers

## Data Availability

The data presented in this study are available on request from the corresponding author.

## Abbreviations

The following abbreviations are used in this manuscript:

UTUC	Upper tract urothelial carcinomas
RNU	Radical nephroureterectomy
KSS	Kidney-sparing surgery
CT	Computed tomography
URS	Ureterorenoscopy
UUT	Upper urinary tract
NBI	Narrow band imaging
PDD	Photodynamic diagnosis
f-URS	Flexible URS
WL	White-light
Se	Sensitivity
Sp	Specificity
CIS	Carcinoma in situ
OCT	Optical coherence tomography
CLE	Confocal laser endomicroscopy
ELUS	Endoluminal ultrasonography
5-ALA	5-aminolevulinic acid
PpIX	Protoporphyrin IX
HAL	Hexaminolevulinate
TDR	Tumor detection rates
AUC	Area under curve
RR	Relative risk
TURBT	Transurethral resection of bladder tumor
FP	False positives
NMIBC	Non-muscle-invasive bladder cancer
PDD-DLA	PDD-guided dual laser ablation
HLA	Ho:YAG laser ablation
PFR	Progression-free survival
RFS	Recurrence-free survival
LG	Low grade
HG	High grade
CSS	Cancer-specific survival
RGB	Red, green, and blue
FO-URS	Fiber-optic URS
CROES	The Clinical Research Office of the Endourology Society
OS	Overall survival
US	Ultrasound
μOCT	Optical attenuation coefficient
octT	OCT-based T staging
